# Branched-chain Amino Acids: Catabolism in Skeletal Muscle and Implications for Muscle and Whole-body Metabolism

**DOI:** 10.3389/fphys.2021.702826

**Published:** 2021-07-20

**Authors:** Gagandeep Mann, Stephen Mora, Glory Madu, Olasunkanmi A. J. Adegoke

**Affiliations:** Muscle Health Research Centre, School of Kinesiology and Health Science, York University, Toronto, ON, Canada

**Keywords:** branched-chain amino acids, skeletal muscle, catabolism, mTORC1, protein synthesis

## Abstract

Branched-chain amino acids (BCAAs) are critical for skeletal muscle and whole-body anabolism and energy homeostasis. They also serve as signaling molecules, for example, being able to activate mammalian/mechanistic target of rapamycin complex 1 (mTORC1). This has implication for macronutrient metabolism. However, elevated circulating levels of BCAAs and of their ketoacids as well as impaired catabolism of these amino acids (AAs) are implicated in the development of insulin resistance and its sequelae, including type 2 diabetes, cardiovascular disease, and of some cancers, although other studies indicate supplements of these AAs may help in the management of some chronic diseases. Here, we first reviewed the catabolism of these AAs especially in skeletal muscle as this tissue contributes the most to whole body disposal of the BCAA. We then reviewed emerging mechanisms of control of enzymes involved in regulating BCAA catabolism. Such mechanisms include regulation of their abundance by microRNA and by post translational modifications such as phosphorylation, acetylation, and ubiquitination. We also reviewed implications of impaired metabolism of BCAA for muscle and whole-body metabolism. We comment on outstanding questions in the regulation of catabolism of these AAs, including regulation of the abundance and post-transcriptional/post-translational modification of enzymes that regulate BCAA catabolism, as well the impact of circadian rhythm, age and mTORC1 on these enzymes. Answers to such questions may facilitate emergence of treatment/management options that can help patients suffering from chronic diseases linked to impaired metabolism of the BCAAs.

## Introduction

Branched-chain amino acids (BCAAs; leucine, isoleucine, and valine) are a special class of amino acids (AA). In addition to being used as substrates for protein synthesis, they can stimulate skeletal muscle protein synthesis (Yoshizawa, 2004; Norton and Layman, 2006; Kamei et al., 2020) and suppress proteolysis (Béchet et al., 2005; Kamei et al., 2020). They also promote glucose transport (Nishitani et al., 2005; Yoon, 2016; Crossland et al., 2020) and have been linked to the regulation of body weight (She et al., 2007a; Siddik and Shin, 2019). In addition, 3-hydroxy-3-methylglutaryl-CoA, a product of leucine catabolism, can be used as a substrate in cholesterol synthesis (Mathias et al., 1981; Zhang et al., 2007) and therefore is important in membrane integrity and cellular communication. In spite of these roles, sustained elevations of the BCAAs in the plasma and skeletal muscle are associated with insulin resistance (Newgard et al., 2009) and type 2 diabetes mellitus (T2DM) (Flores-Guerrero et al., 2018).

**FIGURE 1:**
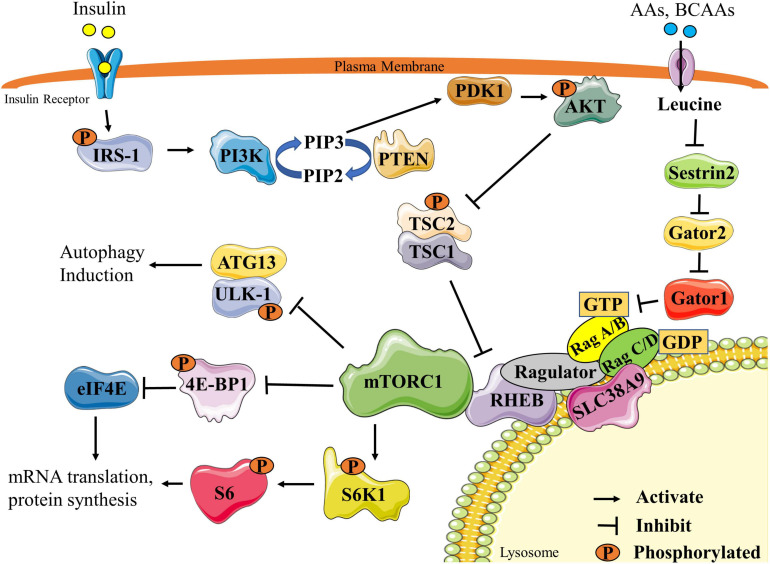
Schematic representation of mTORC1 signaling pathway. Stimulation by insulin ultimately leads to the activation of the PI3K pathway and PIP3 synthesis. PIP3 activates PDK1, which can then phosphorylate AKT. Activated AKT phosphorylates and inactivates the TSC2/1 complex, allowing RHEB to remain GTP loaded to activate mTORC1. Activated mTORC1 activates translation machinery and protein synthesis by phosphorylating two main downstream targets, S6K1 and 4E-BP1. mTORC1 also inhibits autophagy by phosphorylating the ULK1/ATG13 complex. mTORC1 can also be activated through AA or BCAA stimulation of the sestrins/gator/RAG/ragulator pathway. Leucine, the most commonly studied BCAA, inhibits sestrin-2 leading to eventual GTP loading and RHEB activation of mTORC1. Re-drawn and modified from Fan et al. (2017) and Wolfson and Sabatini (2017). 4E-BP1, eukaryotic mRNA translation initiation factor 4E-binding protein 1; AA, amino acids; ATG13, autophagy-related protein 13; BCAA, branched-chain amino acids; eIF4E, eukaryotic mRNA translation initiation factor 4E; IRS1, insulin receptor substrate 1; mTORC1, mammalian/mechanistic target of rapamycin complex1; PDK1, 3-phosphoinositide-dependent protein kinase-1; PI3K, phosphatidylinositol-3 kinase; PIP2, phosphatidylinositol (4,5)-bisphosphate; PIP3, phosphatidylinositol (3,4,5)-trisphosphate; PTEN, phosphatase and tensin homolog; RHEB, Ras homolog enriched in brain; S6K1, ribosomal protein S6 kinase; S6, ribosomal protein S6; SLC38A9, solute carrier family 38 member 9; TSC1/2, Tuberous sclerosis proteins 1 and 2; ULK-1 unc-51 like autophagy activating kinase.

The anabolic effect of the BCAAs, especially of leucine, are mediated in part through the activation of the mammalian/mechanistic target of rapamycin complex 1 (mTORC1) (Figure 1) (Gran and Cameron-Smith, 2011). mTORC1 is a serine/threonine kinase complex that is critical in promoting and maintaining muscle mass (Adegoke et al., 2012). Activation of mTORC1 is triggered by a number of factors, including nutrition [especially BCAAs and other AAs (Bar-Peled and Sabatini, 2014; Moberg et al., 2016), glucose (Kwon et al., 2004), and fatty acids (Yasuda et al., 2014)], growth factors [insulin, insulin-like growth factor 1 (IGF-1)] (Yoon, 2017), energy (Bond, 2016), oxygen status (Cam et al., 2010), statins (Henriksbo et al., 2020) and/or resistance exercise (Adegoke et al., 2012). Full activation of mTORC1 in response to nutrition requires two components. First, insulin/IGF1 induces the activation of the insulin receptor substrate 1 (IRS-1)/phosphatidylinositol-3 kinase (PI3K)/protein kinase B (AKT) pathway, leading to GTP-loading of the mTOR activator Rheb (Hemmings and Restuccia, 2012; Hassan et al., 2013). Second, full activation of mTORC1 requires mTORC1 sensing of the BCAAs via many mediators, the best understood of which is the sestrins/gator/RAG/ragulator pathway (Chantranupong et al., 2014). Activation of these components ultimately leads to the transfer of mTORC1 to the lysosomal membrane where activated Rheb is localized. Once activated, mTORC1 phosphorylates many downstream targets, of which ribosomal protein S6 kinase (S6K1) and eukaryotic mRNA translation initiation factor 4E-binding protein 1 (4E-BP1) are the most studied. Activation of mTORC1 and subsequent phosphorylation of downstream targets stimulates protein synthesis, leading to increases in skeletal muscle fiber size and mass (Bodine et al., 2001b).

mTORC1 also inhibits skeletal muscle proteolysis. Activation of either the ubiquitin proteasome pathway (UPP) and/or autophagy/lysosomal pathways leads to skeletal muscle protein breakdown (Bodine et al., 2001a). Upon activation by the BCAAs, mTORC1 can inhibit signaling events involved in protein breakdown through multiple mechanisms, one of which is the suppression of autophagy via the phosphorylation and inhibition of a number of autophagy regulators, including Unc-51 like autophagy activating kinase (ULK-1) (Hosokawa et al., 2009), transcription factor EB (TFEB) (Martina et al., 2012), Beclin-1-regulated autophagy (AMBRA1) (Nazio et al., 2013) and autophagy-related protein 13 (ATG13) (Hosokawa et al., 2009). Leucine can also suppress proteolysis by suppressing the UPP (Nakashima et al., 2005).

Here, we review the catabolism of BCAAs in skeletal muscle (with reference to other tissues where relevant), and the impact of the BCAAs and their metabolites on skeletal muscle, whole-body metabolism, energy production and disease. Lastly, we comment on outstanding questions that need to be investigated, including mechanisms of regulation of the abundance of enzymes involved in BCAA catabolism, the effects of post translational modifications on the activities of these enzymes in skeletal muscle, effect of age on BCAA catabolism, and the role of mTORC1 in the regulation of BCAA catabolism.

## BCAA Catabolism

**FIGURE 2:**
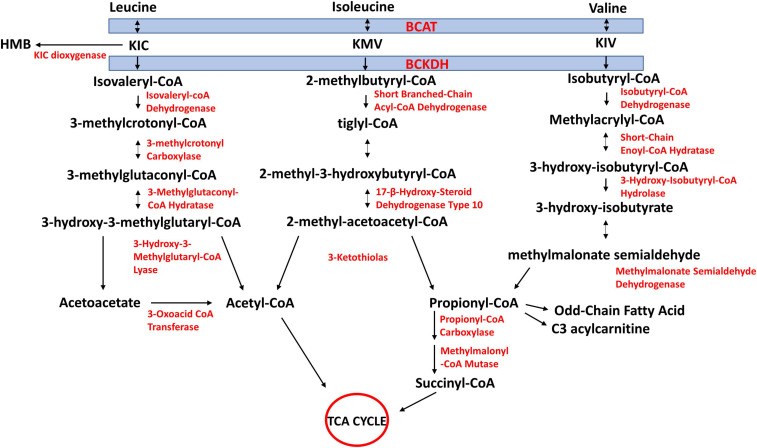
Schematic of branched-chain amino acid (BCAA) catabolism. BCAAs share the same first two steps in their catabolism. They undergo reversible transamination, catalyzed by mitochondrial or cytosolic isoforms of branched-chain aminotransferase (BCAT). Branched-chain ketoacids (BCKAs) produced from this reaction are irreversibly decarboxylated to yield respective CoA compounds, which divide into their respective metabolic pathways. KIC, 2-keto-isocaproate/4-methyl-2-oxopentanoic acid; KMV, α-keto-β-methylvaleric acid/3-methyl-2-oxopentanoate; KIV, 2-keto-isovalerate/3-methyl-2-oxobutanoic acid. Re-drawn and modified from Adeva-Andany et al. (2017).

The first two steps of BCAA catabolism are shared amongst the three BCAAs (Figure 2). These are transamination catalyzed by branched-chain aminotransferase (BCAT) and oxidative decarboxylation catalyzed by the branched-chain α-keto acid dehydrogenase complex (BCKDH). The reversible transamination reactions yield branched-chain α-keto acids [BCKAs; 2-keto-isocaproate/4-methyl-2-oxopentanoic acid (KIC), α-keto-β-methylvaleric acid/3-methyl-2-oxopentanoate, (KMV), and 2-keto-isovalerate/3-methyl-2-oxobutanoic acid (KIV), respectively, from leucine, isoleucine, and valine]. The BCKAs are then irreversibly oxidatively decarboxylated by BCKDH to produce the corresponding acyl CoA derivates (isovaleryl-CoA from KIC, 2-methylbutyryl-CoA from KMV, and isobutyryl-CoA from KIV). The BCKDH reaction is the rate-limiting step in BCAA catabolism and is therefore tightly regulated (Harper et al., 1984; Harris et al., 1990). Beyond this step, the acyl-CoA derivatives are metabolized along separate pathways. Ultimately, leucine catabolism produces acetoacetate and acetyl-CoA, isoleucine yields propionyl-CoA and acetyl-CoA, and valine yields propionyl-CoA (Figure 2). Because high circulating concentrations of BCAAs, which may arise from impaired catabolism of the AAs, have been linked to insulin resistance, T2DM (Chen and Yang, 2015), and cardiovascular diseases (White and Newgard, 2019), an understanding of mechanisms of regulation of tissue catabolism of BCAAs is required for a better understanding of these diseases and how to manage/prevent them.

**FIGURE 3:**
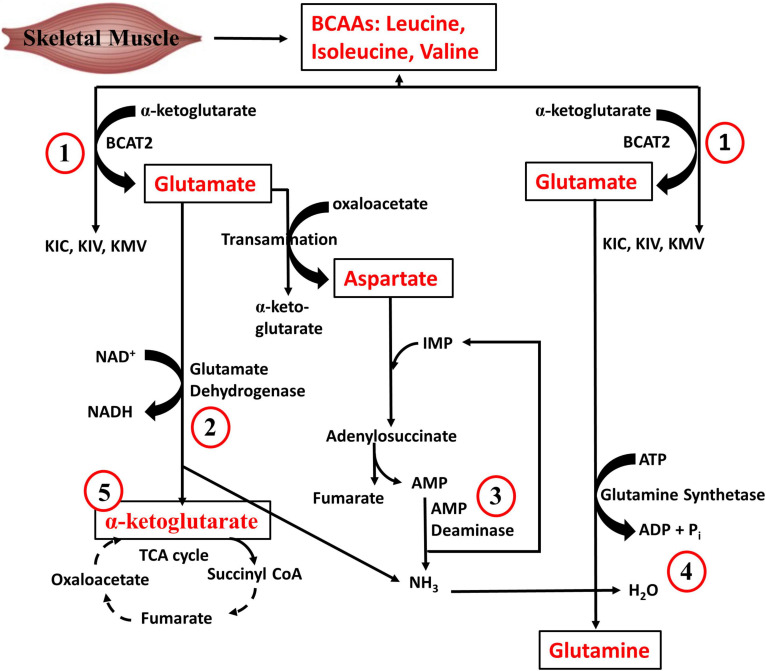
Branched-chain amino acid metabolism in skeletal muscle. (1) BCAAs are transaminated with α-ketoglutarate by BCAA transaminase to generate glutamate. (2) Glutamate deamination yields α-ketoglutarate and ammonia. (3) During exercise, AMP is generated from ATP degradation in the skeletal muscle. The muscle AMP deaminase reaction also forms ammonia. (4) Glutamine is formed from ammonia and glutamate, a reaction catalyzed by glutamine synthetase. (5) The α-ketoglutarate formed by glutamate dehydrogenase can anaplerotically enter the TCA cycle. Re-drawn and modified from Groper and Smith, (2013).

Regarding quantitative tissue contribution to BCAA metabolism, the largest contributors of isoleucine disposal into protein synthesis are the liver (27%), skeletal muscle (24%), pancreas (24%), followed by the kidneys (9%), brown adipose tissue (6%) and other tissues (10%) (Neinast et al., 2018). The pancreas has the highest fractional synthesis rate of protein compared to other tissues (Neinast et al., 2018), which could explain why it is a major contributor to whole body disposal of isoleucine as well as the other BCAAs into proteins. On the other hand, skeletal muscle is the largest contributor to whole-body BCAA oxidation (59%), followed by brown adipose tissue (19%), liver (8%), kidneys (5%), heart (4%), and other tissues (5%). The relative predominance of skeletal muscle in BCAA catabolism is related at least in part to the fact that BCAA transamination, the first step of BCAA catabolism, occurs largely (65%) in the skeletal muscle (Suryawan et al., 1998). In addition to its contribution to BCAA oxidation, muscle metabolism of BCAAs is vital in whole body AA metabolism. As depicted in Figure 3, BCAA-derived ammonia, via glutamate dehydrogenase and glutamine synthetase reactions, is ultimately funneled into glutamine, an AA with roles in many vital body processes. The muscle AMP deaminase reaction (Figure 3), especially during exercise, is also a source of ammonia that can be used to make glutamine. In addition to the ATP that can be generated from the complete oxidation of BCAA to CO_2_, it is also evident from Figure 3 that muscle BCAA catabolism contributes to energy production via the NADH generated from the glutamate dehydrogenase reacction and the anaplerotic supply of α-ketoglutarate into the TCA cycle. The significance of skeletal muscle in whole body BCAA catabolism is emphasized by the fact that even though insulin infusion or inhibition of BCKDH kinase, the enzyme that inhibits BCKDH (see below), increases whole body BCAA oxidation in healthy animals, this is largely driven by the increase in muscle BCAA oxidation. The significance of muscle is even more evident in insulin resistance state: in high fat diet (HFD) fed mice or db/db mice, although BCAA oxidation is decreased by up to 60% in tissues like liver and adipose tissue, BCAA oxidation in skeletal muscle either remained unchanged or increased ∼50% (Neinast et al., 2018). Therefore, our focus is the regulation of BCAA catabolism in skeletal muscle, although appropriate references will be made to other tissues.

### Branched-Chain Aminotransferases (BCAT)

**FIGURE 4:**
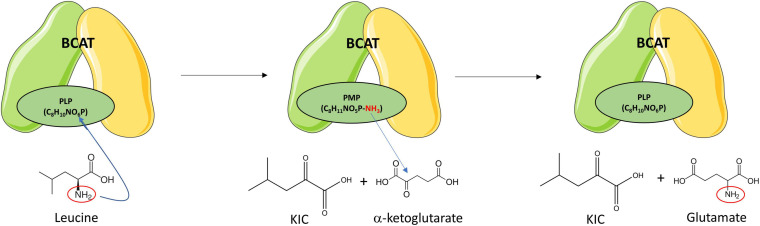
Branched-chain amino transferase reaction. BCAT catalyzes the reversible transamination of branched-chain amino acids (BCAAs) into their respective branched-chain ketoacids (BCKAs). Leucine is transaminated to ketoisocaproic acid (KIC), isoleucine to keto-beta-methylvaleric acid (KMV), and valine to ketoisovaleric acid (KIV). The α-amino group of the BCAA is transferred to BCAT-PLP yielding BCAT-pyridoxamine phosphate (PMP) and the respective BCKA. The α-amino group is then transferred from the BCAT-PMP to α-ketoglutarate producing glutamate and restoring BCAT-pyridoxal phosphate (PLP). Leucine transamination is shown as representative of transamination reactions of the other BCAAs. Re-drawn and modified from Conway and Hutson (2016).

Branched-chain aminotransferases catalyzes the reversible transamination of BCAAs into their respective BCKAs (Figure 2). In one direction, when BCAAs are transaminated to their respective BCKAs, α-ketoglutarate (α-KG) receives the amino group producing glutamate. In the opposite direction, when the BCAAs are produced from their respective BCKAs, glutamate donates the amino group and is converted to α-KG. In the transamination reaction, BCAT requires a coenzyme form of vitamin B6, pyridoxal 5′-phosphate (PLP), that serves as the amino group carrier. The transamination reaction is accompanied by the interconversion between the PLP- and the pyridoxamine 5′-phosphate (PMP)-bound forms of the enzyme. PMP transfers the amino group to α-KG to produce glutamate, restoring BCAT-PLP conformation (Figure 4) (Goto et al., 2005).

There are two isoenzymes of BCAT, cytosolic and mitochondrial forms. The *BCAT1* gene encodes the cytosolic BCAT (BCAT1), while the *BCAT2* gene encodes the mitochondrial BCAT (BCAT2) (Bledsoe et al., 1997). The mitochondrial isoform is much more widespread, being found in skeletal muscle, kidney, cortex, heart, subcutaneous adipose tissue, stomach, colon, ileum, and liver. BCAT1 is restricted to the brain, ovary and placenta (Bledsoe et al., 1997). Both isoforms are not present in the same tissue (Sweatt et al., 2004). BCAT2 is most abundant in skeletal muscle, followed by the kidney, and is least abundant in the liver (Suryawan et al., 1998). Substrate preferences for BCAT proteins is isoleucine, leucine and then valine (Wallin et al., 1990; Hall et al., 1993; Davoodi et al., 1998). Due to the low levels of BCAT2 in the liver, BCAAs are often not metabolized in this tissue but BCKAs arising from the transamination of BCAAs in other tissues can travel to the liver where they can serve as substrates for BCKDH (Kainulainen et al., 2013). Here (see section “BCAT2 and Its Regulation in Skeletal Muscle” below), we will focus on BCAT2, the mitochondrial isoform whose expression is high in skeletal muscle, the tissue where over half the whole body activity of BCAT2 is found (Table 1) (Suryawan et al., 1998).

**TABLE 1:** Relative distribution of BCAT activity and BCAA transamination in human tissues.

Tissue	BCAT activity (mU/g)	Distribution of BCAA transamination
Skeletal muscle	124 ± 14	65.4%
Kidney	880 ± 48	3.8%
Stomach	241 ± 11	7.7%
Liver	248 ± 32	3.8%
Brain	510 ± 49	15.4%
Heart	387 ± 23	N/A
Pancreas	N/A	N/A
Colon	254 ± 23	N/A
Adipose tissue	84 ± 4	N/A

*Table re-drawn and modified from Suryawan et al. (1998). BCAT, branched chain aminotransferase; BCAA, branched-chain amino acids; g, grams of tissue; mU, milliunits; N/A, not applicable.*

### Branched-Chain Ketoacid Dehydrogenase (BCKDH)

**FIGURE 5:**
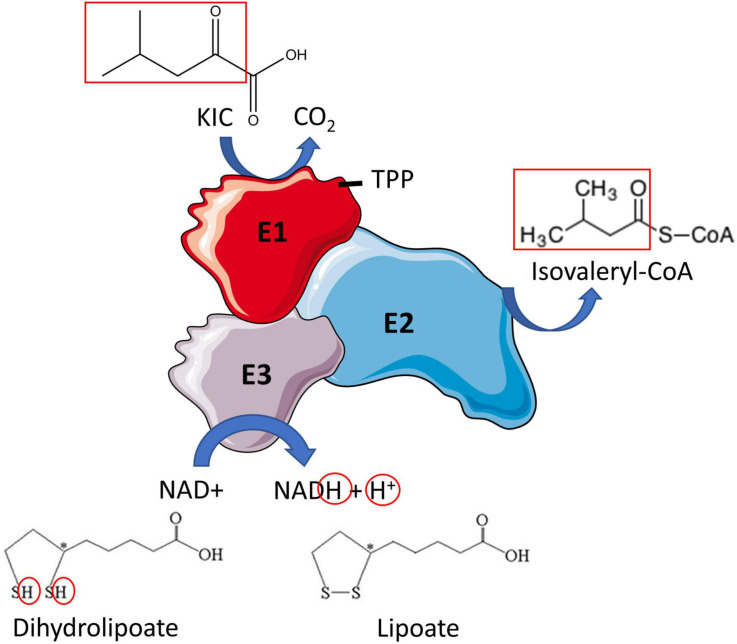
Branched-chain ketoacid dehydrogenase reaction. Oxidative decarboxylation of the branched-chain ketoacid (BCKA) (shown here for ketoisocaproic acid) is initiated when the E1 subunit of BCKDH binds thiamine pyrophosphate (TPP). An acyl group is formed (both red boxes) which is simultaneously transferred to E2, where it is attached to coenzyme A (CoA). Lipoate is oxidized in the process, producing dihydrolipoate. E3 reduces dihydrolipoate producing lipoate and NADH + H^+^. Then the metabolites of the BCKAs are metabolized in their respective pathways. Re-drawn and modified from Conway and Hutson (2016).

Branched-chain ketoacid dehydrogenase catalyzes the irreversible oxidative decarboxylation of BCKAs (Figures 2, 5), producing the respective branched-chain acyl-CoA derivates (isovaleryl-CoA from KIC, 2-methylbutyryl-CoA from KMV and isobutyryl-CoA from KIV) along with CO_2_ and NADH. The BCKDH complex consists of three subunits, heterodimeric branched-chain α-keto acid decarboxylase (E1), dihyrolipoyl transacyclase (E2) and homodimeric dihydrolipoyl dehydrogenase (E3). The E1 subunit is a tetramer comprised of two α and two β subunits (α2β2) and is organized in a tetrahedral arrangement of the two α and two β subunits. The α subunits of E1 are encoded by *BCKDHA* gene and the β subunits by *BCKDHB* gene. Between the α and β subunits are two thiamine-binding pockets, which allow E1 to bind thiamine pyrophosphate (TPP) (Indo et al., 1987) (Figure 5).

The E2 subunit is encoded by *dihydrolipoamide branched-chain transacyclase* gene (DBT) and is the subunit to which E1 and E3 subunits are attached at the center of the complex. The E2 subunit has three domains: the core domain, the binding domain and the lipoyl domain. The core domain has the active site of the enzyme while the binding domains are responsible for the attachment of the E1 and E3 subunits (Conway and Hutson, 2016) through ionic interactions (Ananieva and Conway, 2020). The lipoyl domain is for substrate channeling within the complex (Chuang et al., 2006). The E3 subunit is encoded by *dihydrolipoamide dehydrogenase* (DLD) gene and is a homodimeric flavoprotein that contains a bound flavin adenine dinucleotide molecule (Litwer et al., 1989).

BCKDH reaction occurs in multiple steps. Firstly, the E1 subunit catalyzes the decarboxylation of the BCKA, releasing CO_2_ and the corresponding branched-chain acyl intermediate. This branched-chain acyl group is then transferred by the lipoyl domain of the E2 subunit to the core of the complex where it is attached to coenzyme A by the catalytic domain of E2, producing branched-chain acyl-CoA ester, and lipoate is reduced to dihydrolipoic acid in the process. E3 then reoxidizes the dihydrolipoic acid using NAD+ and produces NADH and lipoic acid (Adeva-Andany et al., 2017). Valine-derived KIV is the preferred substrate for BCKDH in cultured fibroblasts (Yoshida et al., 1986), but no data is available in skeletal muscle. In rat tissues, the BCKDH activity is highest in the liver, intermediate activity in the heart and kidney and is lowest in skeletal muscle, adipose tissue, and brain (Harper et al., 1984). In rats, 83% of BCKD’s oxidative capacity is in the liver, with 3% in skeletal muscle, 10% in the kidney, 1% in the brain, and 2% in the stomach and intestine. The distribution of BCKD oxidative capacity differs greatly in humans, as 54% is in skeletal muscle, 13% in the liver, 8% in the kidney, 20% in the brain, and 4% in the stomach and intestine (Table 2) (Suryawan et al., 1998).

**TABLE 2:** Relative distribution of BCKD activity and BCKD oxidative capacity in human tissues.

Tissue	Distribution of BCKD activity (mU/g)	Distribution of BCKD oxidative Capacity
Skeletal muscle	1.3 ± 0.3	54%
Kidney	15.9 ± 2.6	8%
Stomach + intestine	2.5 ± 0.1	4%
Liver	4.2 ± 0.41	13%
Brain	6.4 ± 0.7	20%
Heart	3.3 ± 0.5	N/A
Pancreas	N/A	N/A
Colon	2.3 ± 0.2	N/A
Adipose tissue	1.1 ± 0.1	N/A

*Table re-drawn and modified from Suryawan et al. (1998). BCKD, branched-chain ketoacid dehydrogenase; BCAA, branched-chain amino acids; g, grams of tissue; mU, milliunits; N/A, not applicable.*

Apart from the BCKDH reaction, BCKAs can have alternative fates. They can be reduced at the α-carbon, forming branched chain α-hydroxy ketoacids in a number of metabolic disorders including maple syrup urine disease (MSUD), propionic acidemia and in ketoacidosis (Liebich and Först, 1984; Anderson et al., 2017). These are present in adult urine at low levels and are directly degraded by α-hydroxyacid oxidases, probably in the liver (Jones et al., 2000). Also, cytosolic dioxygenase, present predominantly in the liver, with a small abundance in the skeletal muscle (Nissen et al., 1996), can convert a small percentage of α-KIC to beta-hydroxy-beta-methylbutyrate (HMB) (Van Koevering and Nissen, 1992) (Figure 2). HMB can increase mTORC1 signaling (Kimura et al., 2014; Suryawan et al., 2020) and muscle protein synthesis (Wilkinson et al., 2013) and suppress proteolysis (Kimura et al., 2014), thus promoting muscle anabolism.

### BCAA Metabolism Downstream of BCKD

Leucine-derived isovaleryl-CoA is ultimately metabolized, via multiple steps, to acetyl CoA and acetoacetate (Figure 2). Acetoacetate can yield two molecules of acetyl CoA. The three acetyl-CoA derived from leucine can enter the TCA cycle or alternate pathways. Similarly, isoleucine-derived 2-methylbutyryl-CoA ultimately yields acetyl-CoA and succinyl-CoA, while the catabolism of valine-derived isobutyryl-CoA yields propionyl-CoA (Adeva-Andany et al., 2017). Whether the catabolism of an AA ultimately leads to the production of only acetyl-CoA/acetoacetate and/or intermediates such as propionyl-/succinyl-CoA that can be converted, via gluconeogenesis, into glucose is the basis of classification of AAs as glucogenic, ketogenic, or both. Because it is generally accepted that acetyl-CoA derived carbon cannot end up in glucose, leucine is the only BCAA that is considered strictly ketogenic; valine is considered glucogenic while isoleucine is both (Figure 2). However, there is still some debate on the fate of acetyl-CoA carbon vis-à-vis gluconeogenesis (Green, 2020; Tetrick and Odle, 2020).

## BCAT2 and Its Regulation in Skeletal Muscle

### Importance of BCAT2 in Muscle

BCAT2 (whole body) knockout (KO) mice are leaner and smaller in size. Knockout mice exhibit reduced endurance in response to exercise (∼70%), and increased muscle S6K1 phosphorylation (∼2-fold) (She et al., 2007a, 2010). These mice also display increased expression of genes associated with protein degradation, apoptosis, and necrosis (Lynch et al., 2015). They have increased plasma BCAA levels (fivefold), which is more remarkable in female animals even when they consumed BCAA-free diets (She et al., 2007a). Blood levels of KMV and KIV are reduced 40–50% in KO animals (She et al., 2007a,b). The increased levels of BCAAs could activate protein synthesis through the activation of mTORC1 (Gran and Cameron-Smith, 2011). Also, the transamination of leucine to KIC is required to inhibit protein degradation in skeletal muscle (Tischler et al., 1982), so a lack of BCAT2 could suppress muscle KIC (which was not measured in the study), which could explain the increase in muscle protein degradation (She et al., 2007a).

BCAT2 KO mice have greater muscle glycogen (∼45%) in a re-fed state after starvation (She et al., 2010). They also have reduced exercise capacity and higher intramuscular lactate/pyruvate ratio (∼1.5-fold), along with reduced intramuscular TCA cycle intermediates (malate and citrate), indicating a potential energy crisis. These observations in BCAT2 knock out mice outline the importance of BCAT2 in skeletal muscle and whole-body metabolism. This data was generated from study of whole-body knockout of BCAT2. It would be interesting to see the effects of muscle specific deletion of BCAT2 on skeletal muscle and whole-body metabolism. Interestingly, myoblasts depleted of BCAT2 are impaired in their ability to form myotubes (Dhanani et al., 2019).

### Physiological Regulation of Muscle BCAT2

#### Exercise

Plasma BCAA levels during exercise (∼67% VO_2max_ for 1 h) and 30 min after exercise do not change in young individuals (19–22 years old) (Poortmans et al., 1974). Refsum et al. (1979) demonstrated 40% decrease in plasma BCAA levels (∼40%) after prolonged exercise (4–6 h of cross-country skiing), in trained individuals aged 23–45 years. In another study, there are significant increases in plasma leucine (10%) and isoleucine (35%) levels 4 h post low-intensity exercise (30% VO2_*max*_), measured in the leg of individuals aged 24–32 years (Ahlborg et al., 1974). Differences in the change in BCAA levels across studies could be attributed to differences in workload intensity and duration. Whole-body leucine oxidation is higher after exercise compared to rest, and leucine oxidation is higher in a fasted state compared to a post absorptive state in human subjects who had exercised. There is no difference in leucine oxidation between starvation and post absorptive state amongst non-exercised controls (Knapik et al., 1991). In the post-absorptive state, there was a greater KIC to leucine ratio after exercise compared to rest (Knapik et al., 1991) suggesting BCAT2 activity could be upregulated. Fielding et al. (1986) support this as intramuscular KIC levels increased during exercise, suggesting an increase in leucine transamination.

Most studies attribute changes in BCAA metabolism after exercise with changes in BCKDH activity (Wagenmakers et al., 1989; Shimomura et al., 1995; Fujii et al., 1998), but there is evidence that BCAT2 too can be regulated by exercise. Roberson et al. demonstrated that BCAT2 protein expression is higher after endurance training compared to resistance training but normalized to similar levels after 3 days of repetitive exercise training (Roberson et al., 2018). They did not measure if this correlates with increased BCAA metabolism, but it is consistent with the literature as endurance exercise increases BCAA oxidation (Wagenmakers et al., 1989) and leucine oxidation (Mazzulla et al., 2017) while resistance exercise does not (Tarnopolsky et al., 1991). In another study, mice underwent bilateral synergist ablation (mechanical overload), removing the gastrocnemius and soleus to induce hypertrophy in the plantaris muscle for up to 14 days. BCAT2 mRNA expression was downregulated (0.48-fold) days 3–7 of mechanical overload in the plantaris. The authors suggested that reduced BCAT2 levels increase BCAA levels (Chaillou et al., 2013), which could in turn activate mTORC1, leading to muscle hypertrophy.

#### Diabetes/Insulin Resistance

Skeletal muscle BCAT2 mRNA level is reduced by 25% in type 2 diabetes patients compared to body mass index-matched controls (Hernández-Alvarez et al., 2017). However, muscle BCAT2 protein level is increased (∼50%), but without a change in BCAT2 mRNA expression in db/db mice, a T2DM mouse model (Hernández-Alvarez et al., 2017). On the other hand, in another study, db/db mice exhibit increased plasma levels of valine (∼50%), suggesting reduced BCAT2 activity (Neinast et al., 2018). In ob/ob mice, an obese mouse model, there is no change in gastrocnemius muscle BCAT2 mRNA or protein levels compared to control, irrespective of whether the animals were fasted or fed (Hernández-Alvarez et al., 2017). In another study, gastrocnemius muscle BCAT2 protein level is not different between lean and obese Zucker rats, irrespective of their nutritional status (She et al., 2007b). Finally, in rats fed fructose for 45 days to produce a non-obese insulin resistance state, there were reductions in muscle BCAT2 activity (15%), even though activities of the enzyme in liver and adipose tissue were not affected (David et al., 2019).

Interestingly, supraphysiological supplementation of metformin (2 mM), a commonly prescribed drug for T2DM, for 12 h increases BCAT2 mRNA expression levels (30%) but significantly reduces it after 24 h in C2C12 myotubes. Protein levels of BCAT2 were also decreased (25%) after 24 h of treatment (Rivera et al., 2020b). Thus, elevation in circulating BCAAs in obesity/insulin resistance states cannot be totally explained by changes in the expression of BCAT2. Although not consistent across all reports, the studies reviewed suggest a reduction in BCAT2 level in diabetes/insulin resistance. The discordance may relate to study models and/or length of study. For example, it is conceivable that in studies with prolonged duration, elevated BCAA levels may induce BCAT2 expression in an effort to reduce the levels of these AAs via increased transamination. Furthermore, increased BCAT2 abundance can regulate BCKDH function (Islam et al., 2010).

Similarly, BCAAs are increased fivefold in animals with type 1 diabetes mellitus (T1DM) (Hutson and Harper, 1981; Aftring et al., 1988; Rodríguez et al., 1997), reviewed in Holeček (2020). This could be due to the significant reduction in BCAT2 mRNA levels in skeletal muscle and liver of rabbits with T1DM induced by alloxan (Gürke et al., 2015). Although insulin is a potent activator of overall muscle and whole body BCAA oxidation (Neinast et al., 2018), much of the work on BCAA metabolism has focussed on insulin resistance/T2DM. Thus, more studies are required to assess the effect of T1DM on BCAT2 and BCAA catabolism, and whether changes in BCAA metabolism in T1DM are linked to the pathologies or complications of the disease.

#### Nutritional Regulation

Valine supplementation in C2C12 muscle cells has no effect on BCAT2 mRNA or protein expression, whether or not insulin resistance was present (Rivera et al., 2020a). In adipocytes, leucine supplementation on day 4 of differentiation increases BCAT2 mRNA levels (twofold), but on day 10 of differentiation, it reduces BCAT2 protein levels (Kitsy et al., 2014). As mentioned, dexamethasone had no effect on BCAT2 mRNA levels, but dexamethasone and 5 mM of leucine (but not 10 mM) supplementation increases BCAT2 mRNA (100%) in C2C12 myoblasts (Wang et al., 2016).

Compared to a diet that contains 17% protein, a diet with 30% protein has no effect on rat muscle BCAT2 mRNA (Cheon and Lim, 2015). On the other hand, a HFD has a more robust effect on BCAT2 expression (Liu et al., 2017). Rats on a HFD for 24 and 32 weeks display increased BCAT2 protein expression in skeletal muscle (twofold for 24 weeks, threefold for 32 weeks), and leucine supplementation with a HFD further increased BCAT2 expression (25%) (Liu et al., 2017). This correlates with a significant reduction in serum isoleucine and valine levels, but no change in leucine levels. Additionally, a HFD increases serum KIV (50%) and KIC (25%) levels after 32 weeks on an HFD, further suggesting the increase in BCAT2 activity. Interestingly, supplementation of leucine in a HFD at a later time (when hyperglycemia had already developed) attenuated the reduction in insulin sensitivity seen in the HFD group by mechanisms including increase in muscle mitochondrial biogenesis (Liu et al., 2017). This is an interesting finding, as obesity/T2DM exhibit increased BCAAs and reduced BCAA catabolism (Lian et al., 2015). While the finding of beneficial effects of leucine supplementation at a later stage of HFD consumption may suggest that timing of the leucine administration is an important factor to consider, it should also be noted that, unlike what is commonly seen in insulin resistance/T2DM, BCAA levels were not increased in the HFD-fed animals studied by Liu et al. (2017).

### Mechanisms of Effects of Physiological/Disease States on BCAT2

The effects of physiological and disease states on BCAT2 and BCAA metabolism may be mediated by one or more of the known regulators of metabolism. For example, mice overexpressing peroxisome proliferator-activated receptor gamma coactivator-1alpha (PGC-1α) in skeletal muscle have increased BCAA catabolism (Hara et al., 1987; Hall et al., 1993) and BCAT2 protein expression (1.25-fold) (Hara et al., 1987), and overexpression of PGC-1α in C2C12 muscle cells significantly elevates BCAT2 mRNA (40%) and protein expression (Hara et al., 1987). Increases in BCAA catabolism in the PGC1α transgenic mice coincides with increased TCA cycle intermediates. The increases in BCAT2 mRNA and protein expression in these transgenic mice could be due to the increased number of mitochondria, as BCAT2 is a mitochondrial enzyme. PGC-1α may also activate BCAT2 via its effects on glucocorticoid receptor (GR), as the GR, working through Kruppel like factor 15 (KLF15), increases BCAT2 expression in skeletal muscle (Tischler et al., 1982; Sahlin, 1985). KLF15 KO mice have reduced (∼30%) skeletal muscle BCAT2 mRNA expression (Islam et al., 2010), while its overexpression increases BCAT2 protein levels in primary skeletal myocytes (Flores-Guerrero et al., 2018). In another study, dexamethasone does not increase BCAT2 mRNA levels in C2C12 myoblasts (Tulp et al., 1979), so more work is required to understand the link between glucocorticoids and BCAT2. Moreover, the supposed link between regulators of mitochondrial abundance and BCAT2 is not always observed. For example, KO of nuclear receptor Rev-erb-α, an activator of mitochondrial biogenesis and function, increases, rather than decreases, muscle BCAT2 mRNA levels (30%) (Marchesini et al., 2003). Similarly, 5-aminolevulinic acid, a compound that activates PGC-1α and mitochondrial content in quadriceps, reduces BCAT2 mRNA expression (80%). Thus, there might be more to BCAT2 regulation than merely PGC-1α expression and oxidative capacity (Espinal et al., 1986).

The effects of diverse physiological states on BCAA metabolism may also be mediated by small metabolites. For example, reduced levels of BCAAs in liver cirrhosis patients occur in parallel with hyperammonemia (Lackey et al., 2013). Ammonia infusions results in decreases in BCAA levels in skeletal muscle. This decline could be because of the requirement to use BCAAs in transamination reactions with α-KG to produce glutamate to facilitate ammonia detoxification in skeletal muscle (Henriksson et al., 1986). The decline in BCAAs to detoxify high ammonia levels is also present in urea cycle disorders (Perng et al., 2014). Similarly, in chronic renal insufficiency BCAA levels are reduced and the authors suggest that these AAs are metabolized to produce glutamine to counter the increase in ammonia (Holeček, 2018), implying that an increase in BCAT2 activity in individuals with liver cirrhosis, urea cycle disorders and chronic renal insufficiency can mitigate the increases in ammonia levels.

### Post-transcriptional/Translational Modifications of BCAT2

Except for the relatively well studied effects of branched-chain ketoacid dehydrogenase kinase (BDK) and PP2Cm on the E1α subunit of BCKDH (please see section “BCKDH Regulation in Muscle”), not much is known about posttranslational modifications of enzymes that catabolize BCAAs. In addition to regulation of its mRNA and protein levels, BCAT2 is subject to other forms of regulation, in particular post translational modifications. Overexpression of the mutant form of the proto-oncogene GTPase KRAS (KRAS^G12V^) in pancreatic ductal adenocarcinoma (PDAC) samples increases, whereas knockdown of KRAS decreases, BCAT2 protein, with a minimal effect on BCAT2 mRNA level. The increased stability of BCAT2 under this condition is due to reduced interaction of BCAT2 with tripartite motif containing-21 (TRIM21), a member of the tripartite motif family that functions as a RING finger E3 ubiquitin protein ligase. This results in reduced ubiquitylation but increased stability of BCAT2. The ubiquitylation and degradation of BCAT2 by TRIM21 requires the phosphorylation of BCAT2 by spleen tyrosine kinase (SYK) on Tyr228 (Li J. T. et al., 2020). In addition, BCAT2 is acetylated on Lys44, a modification that does not affect the activity of the enzyme but rather promotes its ubiquitin-dependent degradation. cAMP-responsive element-binding (CREB)-binding protein (CBP) and sirtuin4, respectively, acetylates and deacetylates BCAT2. Interestingly, BCAA depletion promotes acetylation and ubiquitin dependent degradation of BCAT2 in pancreatic cancer cells (Lei et al., 2020). It will be interesting to see whether these novel mechanisms of regulation of BCAT2 are also observed in skeletal muscle.

In a *Escherichia coli*, BCAT2 is negatively regulated by H_2_O_2_. Two cysteine residues (Cys315 and Cys318) form a disulfide bond under oxidizing conditions, reducing the activity of BCAT2 (Conway et al., 2003). Supplementation of dithiothreitol, an antioxidant compound, completely reversed the oxidation and restored activity of BCAT2 (Davoodi et al., 1998; Conway, 2020). This demonstrates that there is redox-linked regulation of BCAT2 activity but this is yet to demonstrated in mammalian skeletal muscle.

BCAT2 is also regulated by microRNA. MicroRNAs (miRNA) are non-coding short RNA molecules that regulate gene expression (Ambros, 2004). *BCAT2* gene is a translational target of microRNA (miR)-182, which is important in axon outgrowth and dendrite maturation (Wang et al., 2017). BCAT2 expression is downregulated by miR-182 (Wang et al., 2017). miR-330-5p is another negative regulator of BCAT2, as there is an inverse relationship between the two during ovine preadipocyte differentiation (Shi et al., 2018). miR-125a-3p is involved in AA and glucose metabolism, and can inhibit BCAA metabolism by negatively regulating KLF-15, an upstream regulator of BCAT2 in skeletal muscle of fish (Li H. et al., 2020).

Much of what is known about BCAT2 relates to the regulation of its abundance. The recent discovery of the miRNAs that can regulate BCAT2 abundance, and of the ubiquitin protein ligase that regulates acetylation/phosphorylation-dependent ubiquitination and degradation of this protein represents novel mechanisms of regulation of BCAT2 and BCAA oxidation (Lei et al., 2020; Li J. T. et al., 2020). First, there is a need to identify mammalian skeletal muscle expression of these enzymes and how they are regulated. In conditions where BCAT2 expression changes, it will be interesting to assess whether these recently discovered enzymes mediate the change in BCAT2. One would predict that conditions that increase BCAT2 abundance and/or BCAA oxidation, for example increased supply of the BCAA, would lead to reduced phosphorylation, acetylation, and ubiquitination of the enzyme. Secondly, because post translational modifications like ubiquitination (Adegoke et al., 2020) and oxidation (Davoodi et al., 1998; Conway, 2020) can affect enzyme functions/localization, an intriguing question would be whether BCAT2 activity too can be so regulated, a discovery that may explain studies in which, for example, changes in BCAT2 expression do not correspond with changes in BCAA oxidation (She et al., 2007b; Hernández-Alvarez et al., 2017). Because these posttranslational modifications happen relatively quickly and are reversible, they may be used by skeletal muscle for short-term regulation within a time frame during which changes in gene or protein expression might not be apparent.

## BCKDH Regulation In Skeletal Muscle

**FIGURE 6:**
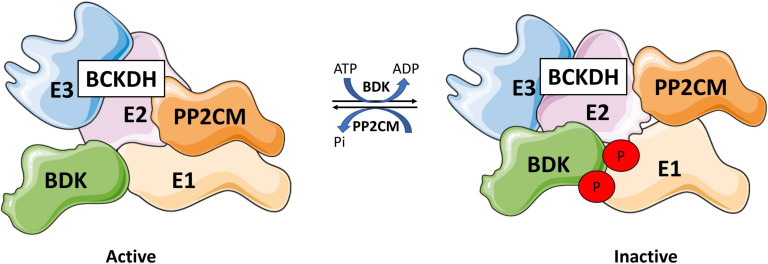
Branched-chain ketoacid dehydrogenase regulation. BCKDH is regulated by a kinase, branched-chain ketoacid dehydrogenase kinase (BDK), and a phosphatase, protein phosphatase 2Cm (PP2Cm) that interact with E1 subunit. BDK phosphorylates BCKDH at Ser293 and 303 inhibiting BCKDH, while PP2Cm dephosphorylates BCDKH, reactivating the complex. Drawn using information from Damuni et al. (1984) and Popov et al. (1991).

BCKDH activity is regulated by a kinase and a phosphatase (Nobukuni et al., 1989). BCKDH kinase (BDK), encoded by the *BCKDK* gene, phosphorylates Ser293 and Ser303 of the E1 subunit of the BCKDH complex and inactivates it (Shimomura et al., 1990; Popov et al., 1991, 1992; Wynn et al., 2000). Conversely, protein phosphatase 2Cm (PP2Cm), encoded by protein phosphatase Mg^2+/^Mn^2+^ dependent 1K (PPM1K), is responsible for the reactivation of the complex by dephosphorylation (Figure 6) (Damuni et al., 1984; Damuni and Reed, 1987). There is also evidence that BCAT2 can physically interact with the E1 subunit of BCKDH, which allows for substrate channeling, increasing the rate of decarboxylation of the complex as a whole (Islam et al., 2007).

The abundance and activity of the BCKDH complex is responsive to nutritional, hormonal, and metabolic conditions. The effects of these conditions are relayed to the BCKDH complex at least in part through phosphorylation/dephosphorylation of the complex. In addition, BCKDH levels are allosterically regulated by branched-chain acyl-CoA esters and NADH, end products of the BCKDH reaction in kidney (Boyer and Odessey, 1991) and liver (Parker and Randle, 1978). Whether these allosterically regulate BCKDH specifically in muscle remains to be seen.

### BDK Structure and Regulation

#### BDK Structure

**FIGURE 7:**
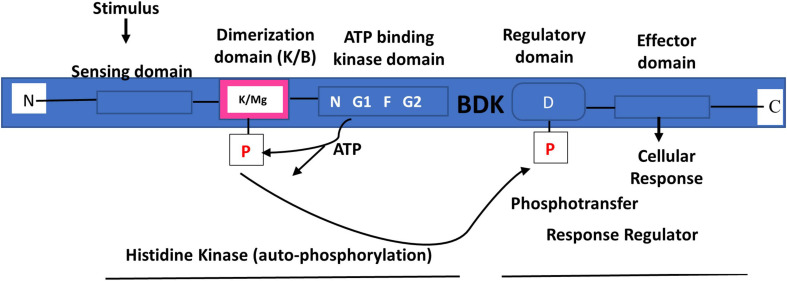
Branched-chain ketoacid dehydrogenase kinase structure and the two-component signal transduction pathway. BDK contains ATP kinase domain with conserved N, G1, F, and G2 motifs. The phosphorylation of BCKDH by BDK is mediated by magnesium (Mg) and potassium (K). Drawn using information from Machius et al. (2001).

BDK is a mitochondrial protein kinase (mPK) that contains a nucleotide-binding domain and a four-helix bundle domain. It is similar in structure to protein histidine kinases (PHKs) (Figure 6) (Popov et al., 1992; Machius et al., 2001). However, unlike PHKs, phosphotransfer carried out by BDK on BCKDH E1α subunit is not mediated by histidine, but by potassium and magnesium (Figure 7) (Machius et al., 2001). In solution, the enzyme can exist as a homodimer (Machius et al., 2001) or tetramer (Wynn et al., 2000; Machius et al., 2001). As a homodimer, it can undergo auto-phosphorylation (Wynn et al., 2000). Changes in the ATP-binding domain attenuates BDK catalytic activity, suggesting that it is of the ATPase kinase superfamily (protein kinases with intrinsic ATPase activity), rather than the PHK family (Wynn et al., 2000). Crystal structure of BDK shows that an allosteric inhibitor such as (S)-α-chloro-phenylpropionic acid [(S)-CPP] binds to a site in the N-terminal domain to cause a movement of the helix-bundle and a conformational change in BDK to promote full activation and dephosphorylation of BCKDH (Figure 7) (Popov et al., 1991; Obayashi et al., 2001; Shimomura et al., 2006; Tso et al., 2013).

#### BDK Regulation

BDK mRNA expression is highest in skeletal muscle, intermediate in the brain and kidney and lowest in the liver and small intestine (Suryawan et al., 1998). Although BDK concentration is lowest in the small intestine, BCKDH complex activity in the small intestine is also low (Suryawan et al., 1998). BDK exists in the mitochondrial matrix of a cell in two forms: a free form and bound form (Popov et al., 1991; Obayashi et al., 2001; Shimomura et al., 2006). A small number of BDK binds BCKDH complex before BDK can be catalytically active and promote the phosphorylation and inactivation of the BCKDH complex. The ketoacids of leucine and valine, KIC and KIV, respectively, induce conformational change and annul the attachment of BDK to the BCKDH complex (Harris et al., 1990). BDK inhibitors such as clofibric acid and thiamine diphosphate (ThDP) also promote the dissociation of BDK from BCKDH complex (Murakami et al., 2005). The inhibition of ThDP is influenced by the physiological concentrations of potassium (Akita et al., 2009).

There is a high ratio of BDK to the E2 subunit of BCKDH in skeletal muscle compared to other tissues, hence the low activity of BCKDH in muscle (Suryawan et al., 1998). The fully lipoylated E2 transacyclase interacts with BDK to promote the phosphorylation of the E1 subunit of BCKDH (Wynn et al., 2000). Maximal decarboxylation activity of BCKDH is determined by the full lipoylation of the E2 subunit of BCKDH (Chuang et al., 2000) Consequently, increased BDK activity decreases the decarboxylation activity and the concentration of the subunits of BCKDH (Chuang et al., 2000).

Mice with muscle-specific deletion of BDK (BDK-mKO) have low skeletal muscle BCAA concentrations, with reduced leucine (∼50%), isoleucine (∼75%), and valine (50%). These mice also exhibit decreased phosphorylation of S6K1 on Thr389 (50%) and of 4E-BP1 (∼25%) when fed a low protein diet (Ishikawa et al., 2017). They have increased BCKDH activity in the heart (20%) and kidney (∼10-fold) (activity in muscle was not measured). They also show a decrease in myofibrillar protein content (∼20%) suggesting that increased BCAA catabolism negatively regulates protein synthesis in skeletal muscle (Ishikawa et al., 2017).

#### Nutritional Regulation of BDK

The activity state of rat hepatic BCKDH complex is regulated by its substrates KIC, KIV, and KMV via their effect on BDK. KIC inhibits BDK and thus activates BCKDH complex in rat liver and heart (Paxton and Harris, 1984; Popov et al., 1991). KIV and KMV also inhibit BDK but with reduced efficiency (Paxton and Harris, 1984; Shimomura et al., 2001). In rat liver, BCAA starvation and low protein diets (8% protein) increase BDK mRNA levels to inactivate the BCKDH complex by more than 90% (Espinal et al., 1986; Popov et al., 1995; Kobayashi et al., 1999; Harris et al., 2001). There is a decreased E1α/E2 ratio with low protein diet (5% leucine) that promotes BCKDH complex sensitivity to phosphorylation-induced inhibition by BDK (Zhao et al., 1994). On the other hand, high glucose levels suppress the activity of the E1α component of BCKDH and the whole complex in the islet of the pancreas (MacDonald et al., 1991). Rats re-fed a high fructose diet (60% fructose) for 4 h after an overnight fast exhibit 75% higher BDK mRNA expression in the liver (White et al., 2018). Because BDK also activates liver lipogenesis by phosphorylating ATP-citrate lyase at Ser454 residue (White et al., 2018), others have studied the effect of HFD on the activity of the enzyme. Rats on a HFD for 24 weeks have reduced (−25%) muscle BDK protein levels but calorie restriction and leucine treatment attenuate this decrease. However, there is no change in BCKDH E1α protein levels. Rats on a HFD for 32 weeks have increased (∼50%) muscle BDK protein levels, consistent with a ∼25% reduction in BCKDH E1α protein levels (Liu et al., 2017). These data correlate with a significant increase in serum KIC (50%) and KIV (25%) after 32 weeks of a HFD, further emphasizing the reduction of BCAA catabolism in a HFD model (Liu et al., 2017).

#### Hormonal Regulation of BDK

There is evidence for hormonal regulation of BDK. Hyperthyroidism, induced by thyroid hormone (T3, 3,5,3′-triiodothyronine) treatment, reduces BCKDH activity in the liver (∼70%), and does so through a 3× increase in BDK activity (Kobayashi et al., 2000). This effect is not seen in skeletal muscle, as BCKDH activity is already low (Kobayashi et al., 2000). Furthermore, as mentioned in Section “Nutritional Regulation of BDK,” protein starvation increases BDK expression, and it also increases serum T3 (Edozien et al., 1978; Tulp et al., 1979), supporting the role of T3 in regulating BDK. Glucocorticoids on the other hand suppresses the expression of BDK. Liver BDK mRNA levels are reduced after dexamethasone treatment in protein starved rats (Huang and Chuang, 1999). Dexamethasone treatment also decreased BDK mRNA levels, increasing BCKDH activity in rat hepatoma H4IIE cells (Huang and Chuang, 1999). Glucocorticoids stimulate gluconeogenesis and since valine and isoleucine are glucogenic, the effect of glucocorticoids may be through the activity of BCKDH, as they downregulate BDK to activate BCKDH to potentially provide more substrate for gluconeogenesis (Harris et al., 2001). One study demonstrated that the glucocorticoid methylprednisolone upregulates BCKDH activity in skeletal muscle, similar to dexamethasone treatment in liver cells, although the effect on BDK was not measured (Block et al., 1987).

Diurnal rhythm and sex hormones may also regulate BDK. BCKDH activity is subject to diurnal rhythm, with activity being low at the beginning of the dark cycle, which also corresponds to increased BDK activity (Kobayashi et al., 1997; Obayashi et al., 2004). This rhythm is observed in females, but not in male rats, which is indicative of regulation of BDK activity by the sex hormone, estrogen (Kobayashi et al., 1999; Obayashi et al., 2004). Indeed, the rhythm is lost in gonadectomized animals (Kobayashi et al., 1999; Harris et al., 2001). Finally, insulin effects a one-fold increase in BDK mRNA expression and a two-fold increase in BDK protein levels in Clone 9 rat cells (Nellis et al., 2002). These data are at odds with other reports that show that insulin increases whole body (Castellino et al., 1987) and skeletal muscle (Neinast et al., 2018) BCAA oxidation. Additionally, as discussed in Section “Diabetes/Insulin Resistance,” T1DM increases BCAA levels, and Aftring et al. (1988) showed that insulin treatment attenuated the increases in plasma and intramuscular BCAAs in diabetic rats. Given that protein metabolism is influenced by sex (Chevalier et al., 2005; Murton and Greenhaff, 2009; Comitato et al., 2015), it is perhaps not surprising that BDK, and by implication, BCAA catabolism can be regulated by sex hormones. Nevertheless, this variable has not been rigorously studied. For example, it would be interesting to examine if alterations to BDK (and related enzymes involved in BCAA catabolism) in insulin resistant/diabetic individuals are sex-dependent, and if such differences are somewhat linked to differences between the sexes in measures of protein metabolism and abnormalities associated with insulin resistance and its sequelae like T2DM and cardiovascular disease. Diurnal regulation of BDK and BCAA catabolism has been even less studied. Because disruption of circadian rhythm is implicated in a number of diseases, including cardiovascular disease, obesity, and some cancers (Scheer et al., 2009; Baron and Reid, 2014; Rijo-Ferreira and Takahashi, 2019), that BDK regulation is under circadian rhythm regulation is of particular interest. Along this line, KLF15 is a transcriptional factor that regulates rhythmicity of AAs (including BCAAs) and nitrogen metabolism (Jeyaraj et al., 2012). Interestingly, a recent study in an animal model of spinal muscular atrophy (SMA) links disruption of circadian rhythm in the regulation of skeletal muscle BCAA catabolism to severity of phenotypes in these animals (Walter et al., 2018). Additional studies are needed to examine whether other enzymes involved in BCAA catabolic pathway, in addition to BDK, are also subject to circadian rhythm regulation, mechanisms of such regulation, and whether such regulation is altered in insulin resistance and its sequelae.

#### Transcriptional/Post-translational Regulation of BDK

There is a carbohydrate response element-binding protein-β (ChREBP-β) binding site 2.5 kb upstream of the BDK transcriptional start site (Jeong et al., 2011). Furthermore, adenovirus ChREBP-β treatment for 7 days upregulates BDK transcripts (∼50%) in rat liver (White et al., 2018). As discussed in Section “Nutritional Regulation of BDK,” BDK is implicated in lipogenesis by activating ATP-citrate lyase. Since ChREBP-β activation contributes to fatty liver and dyslipidemia (Iizuka, 2017), regulation of BDK by this transcription factor further emphasizes the connection between the BCAA catabolic pathway and fatty acid metabolism.

BDK is also regulated post-translationally. Phosphorylation of BDK by Src on Tyr246 enhances BDK activity and stability which promotes metastasis in human colorectal cancer, by enhancing migration, invasion and epithelial to mesenchymal cell transition of colorectal cancer cells (Tian et al., 2020). BDK promotes colorectal and hepatocellular cancers through an alternative pathway that does not involve catabolism of BCAAs. In this context, BDK overexpression increases MEK and ERK activation, which has been linked to carcinogenesis (Lu et al., 2012; Cheng et al., 2016; Xue et al., 2017; Zhai et al., 2020). BDK inhibition by phenyl butyrate reverses this effect (Xue et al., 2017), suggesting that BDK could be a suitable target in the prevention of these cancers. In hepatocellular cancer, Aminopeptidase N is thought to regulate phosphorylation of Ser31 of BDK, which then increases BDK-mediated phosphorylation and activation of ERK1/2, a modification that is associated with increased hepatocellular carcinoma metastasis. However, the specific kinase that phosphorylates BDK on this site has not been elucidated (Zhai et al., 2020). This further emphasizes the importance of BDK in cancer progression and the link between BDK and ERK signaling, a link that has not been investigated in skeletal muscle. Combined with the already discussed effect of BDK on ATP-citrate lyase, this effect of BDK on ERK1/2 signaling demonstrate roles for BDK that appear independent of BCAA catabolism. It also raises the intriguing question of whether signaling pathways that regulate BCAA-independent functions of BDK can regulate this enzyme and whether such a regulation would have a ‘spilling’ effect on BCAA catabolism.

Ubiquitination of BDK is promoted by a ubiquitin E3 protein ligase (UBE3B) and this ubiquitination is disrupted in individuals with Kaufman oculocerebrofacial syndrome. In skeletal muscle of UBE3B KO mice, there is an accumulation of BDK that leads to a decrease in BCKDH complex activity, but this does not correlate with changes in BCAA levels, indicating that BCKAs could be driven to other pathways (Cheon et al., 2019). As discussed above, BDK phosphorylates ATP-citrate lyase, which is crucial for lipogenesis, an important process for neuronal development (Ziegler et al., 2017), therefore BDK accumulation may disrupt the regulation of lipogenesis in developing neurons. The focus of BDK transcriptional/translational regulation of BDK has mostly been in liver so more work on transcriptional/translational modifications of BDK in skeletal muscle is required.

### PP2Cm Structure and Regulation

#### PP2Cm Structure

PP2Cm is a BCKDH phosphatase encoded by the *PPM1K* gene and is highly conserved in vertebrates (Lu et al., 2009; Dolatabad et al., 2019). The phosphatase activity of PP2Cm is dependent on Mn^2+^ bound in the active site (Wynn et al., 2012). PP2Cm is highly expressed in the liver, brain, heart, kidney and diaphragm, but low in skeletal muscle (Zhou et al., 2012). The enzyme binds and dephosphorylates BCKDH complex at Ser293 of the E1α (Lu et al., 2009). Mutation of the Ser293 residue, but not Ser303, affected the interaction of BCKDH with PP2Cm (Lu et al., 2009), suggesting that Ser293 is the critical residue in BCKDH activation. This interaction is disrupted in the presence of BDK suggesting that PP2Cm and BDK compete for interaction with the BCKDH complex (Zhou et al., 2012). Whole body knockout of PP2Cm abrogates the dephosphorylation and increase the hyperphosphorylation of the E1α subunit of BCKDH complex in mice liver and heart (Lu et al., 2009; Wynn et al., 2012; Zhou et al., 2012). Mice lacking PP2Cm show 3–4-fold increases in circulating BCAAs and 5–10-fold increases in liver BCKAs (Abell, 2019), which is associated with numerous diseases, including insulin resistance, obesity, T2DM (Perng et al., 2014; Giesbertz et al., 2015). In liver, PP2Cm also can dephosphorylate ATP-citrate lyase to prevent lipogenesis (White et al., 2018).

#### PP2Cm Regulation

PP2Cm is regulated transcriptionally in response to nutrients (Zhou et al., 2012). Food deprived mice show a decrease in PP2Cm mRNA levels (Lu et al., 2009). BCAAs and BCKAs promote the interaction of PP2Cm with the BCKDH complex (Lu et al., 2009). In HepG2 cells, removal of BCAAs decreases PP2Cm expression (40%) while BCAA replenishment in medium rescues PP2Cm mRNA levels (Zhou et al., 2012). The effect appears to be at the transcriptional level, as PP2Cm promoter activity is decreased by 50% when BCAAs are removed from the medium (starvation) (Zhou et al., 2012). Expression of PP2Cm (mRNA and protein) in rat heart is down-regulated in pressure overload model of heart stress (Lu et al., 2007), which is not what you would expect, as catabolic pathways are induced during stress (Church et al., 2019). Similar to BDK discussed in Section “Transcriptional/Post Translational Regulation of BDK,” PP2Cm is also regulated by ChREBP-β in the liver, but in a negative manner. This results in an upregulation of lipogenesis (White et al., 2018). Also, rats referred a high fructose diet (60% fructose) for 4 h after an overnight fast exhibit lower PP2Cm mRNA expression (25%) (White et al., 2018). High fructose diets induce insulin resistance (Basciano et al., 2005), and reduced PP2Cm is consistent with reduced BCKDH activity seen in insulin resistant states like diabetes, as discussed later in Section “Type 2 Diabetes/Obesity.” The fact that substrate availability and insulin resistant regulate PP2Cm expression are consistent with what one might expect. Unlike BCAT2, BCKDH-E1α subunit, and BDK, for which there is at least a few evidence of posttranslational modifications, we did not see evidence that PP2Cm is so regulated. Furthermore, mechanisms that regulate the abundance of the enzyme are yet to be identified.

##### MicroRNAs

Like BCAT2, PP2Cm too is regulated by miRNA. PPM1K mRNA expression is suppressed by miR-204/211 in mouse cell (NIH 3T3) and miR-22 in human cells (HepG2 and HeLa) (Pan et al., 2015). Overexpression of these miRNAs decreases the 3-UTR′ activity of endogenous mRNA of PPM1K (Otulakowski and Robinson, 1987). Finally, BCKDH mRNA expression is controlled by human miR-29b and miR-222. miR-29b interacts with the mRNA of the E2 subunit of BCKDH complex to prevent its translation in human HEK293 cells (Menkes et al., 1954). Studies on the regulation of BCAA catabolism by miRNA are only at the initial stage: we are unaware of data on muscle miRNA that controls PP2Cm expression. Such regulation presents an attractive option to control the metabolism of these AAs since the expression of miRNA can be upregulated or downregulated to control the abundance of their targets (Peng and Wang, 2018).

### Nutritional and Physiological Regulation of BCAA Metabolism and Related Enzymes

#### Fatty Acid Oxidation

Clofibric acid, a drug that promotes fatty acid oxidation, increases gastrocnemius muscle BCKDH activity by ∼50% (Paul and Adibi, 1980). Peroxisome proliferator-activator receptor-α (PPARα) might mediate the effect of free fatty acids (FFA)/FFA oxidation on BCAA oxidation (Kobayashi et al., 2002). Circulating long-chain FFA released due to starvation and exercise bind to PPARα to activate fatty acid oxidation in skeletal muscle and liver (Minnich et al., 2001). Interestingly, PPARα that is released in response to exercise inhibits hepatic BDK expression and promotes BCKDH activity in skeletal muscle (Brabin et al., 2001; Shimomura et al., 2004b).

#### Exercise

Endurance exercise promotes BCAA catabolism and activation of BCKDH complex in human (Wagenmakers et al., 1989) and rat (Shimomura et al., 1995; Fujii et al., 1998) skeletal muscles via an increase in fatty acid oxidation. Manipulation of the BDK-BCKDH interaction is a potential short-term regulatory mechanism for the activity of BCKDH complex (Xu et al., 2001). Exercise training increases human skeletal muscle BDK protein content by 30% and decreases BCKDH complex activity (Howarth et al., 2007). This could be due to the significant increase in NADH levels during maximal exercise and submaximal isometric contractions (Sahlin, 1985; Henriksson et al., 1986), as NADH inhibits BCKDH activity (Brosnan and Brosnan, 2006). However, most studies show the opposite. Endurance training reduces the number of BDK bound to BCKDH complex in rat liver and skeletal muscle, with a greater reduction in liver (∼71%) compared to skeletal muscle (∼30%) (Fujii et al., 1998; Kobayashi et al., 1999; Xu et al., 2001; Shimomura et al., 2004a). This reflects the low activity state of BCKDH complex in the muscle (Xu et al., 2001). The reduction in BDK levels in response to exercise could partly explain increased BCKDH activity after exercise but increases in KIC concentration too could mediate the effect of exercise on BDK and BCKDH activity because of the inhibitory effect of KIC on BDK. This is in line with the observation of an increase in KIC concentration in response to electrically stimulated muscle contraction (Shimomura et al., 1993). BCAA catabolism may be increased due to the reduction in glutamine levels post exercise. Glutamine synthesis is an ATP-dependent condensation of glutamate and ammonia. Glutamate and glutamine levels and glutamine synthesis are reduced post-exercise while glutamine uptake is unchanged in rat skeletal muscle (Dos Santos et al., 2009). Additionally, plasma ammonia levels are increased post exercise (Chen et al., 2020). Thus, it is possible that increased BCAT activity is needed to produce glutamate for ammonia detoxification (Holecek, 2013). BCAA catabolism also feeds into the TCA cycle to ultimately produce ATP. Therefore, BCAA catabolism could be enhanced to replenish the glutamate and ATP pool for glutamine synthesis/ammonia detoxification post exercise. During exercise, there is a net breakdown of proteins to produce AAs for oxidation and gluconeogenesis (Lynis Dohm et al., 1987). This could also partly explain the increase in BCAA catabolism during exercise.

#### Adiponectin

Adiponectin (APN) is an adipokine that regulates glucose metabolism and fatty acid breakdown (Turer and Scherer, 2012). There is evidence that it can regulate BCAA catabolism. APN whole-body KO mice fed HFD have reduced (50%) PP2Cm expression and BCKDH activity in liver and adipose tissue. In addition, these mice exhibit ∼50% increases in liver, adipose tissue and skeletal muscle BDK mRNA expression, as well as elevated plasma BCAA (40%) and BCKA (∼30%) levels (Lian et al., 2015). APN treatment reverses these effects. The effect of APN on BCKDH activity is mediated by PP2Cm, as PP2Cm deficiency partially inhibits APN-induced activation of BCKDH. This effect of APN on BCKDH was also abolished when AMPK was inhibited. Supplementation of AMPK activator, AICAR, decreased liver, adipose tissue, and skeletal muscle BDK protein abundance, and increased PP2Cm protein abundance in adipose tissue and liver. AICAR supplementation also increased BCKDH activity in skeletal muscle (Lian et al., 2015). Another study supports this as APN treatment corrects increases in BCAA concentrations from a HFD in skeletal muscle (Liu et al., 2013). APN is reduced (∼25%) in obesity and diabetes (Weyer et al., 2001), conditions that are also associated with reduced liver, adipose tissue and skeletal muscle PP2Cm and BCKDH activity (Lian et al., 2015; Biswas et al., 2019). Adiponectin regulation of BCAA metabolism adds to the connection between BCAA catabolism, fatty acid oxidation and adipose tissue regulation of whole body insulin sensitivity via regulation of BCAA catabolism.

## Effect of Different Diseases on BCAA Catabolism in Skeletal Muscle

Skeletal muscle is a major site for the initial step of BCAA catabolism, leading to the release of alanine and glutamine in the blood (Holeček, 2018). There are links between BCAA catabolism and disorders such as MSUD, isovaleric acidemia (IVA), methylmalonic acidemia (MMA), propionic acidemia (PA), liver cirrhosis, sepsis, chronic renal failure (CRF), muscle wasting, type 2 diabetics/obesity and neurodegenerative disorders like Alzheimer’s disease (AD) (Burrage et al., 2014; Manoli and Venditti, 2016; Siddik and Shin, 2019). In this section we will discuss how these diseases affect muscle BCAA catabolism. While other diseases too may affect BCAA catabolism, we have focussed on those for reason of space and because there is sufficient data to support the link between these conditions and BCAA catabolism. In the next section, we discuss the effects of altered muscle BCAA catabolism on whole body metabolism.

### Liver Cirrhosis

Reductions in both plasma BCAAs and protein synthesis are seen in liver cirrhosis patients (Holecek et al., 2011). Mechanistically, decreased BCAAs in this condition may be due to an enhancement of BCKDH complex activity (Shimomura et al., 2006). Reduced BCAA concentrations resulting from elevated BCKDH activity suggest that BCAA supplementation may be beneficial for patients. Indeed, 6 months of daily BCAA consumption (12.45 g of BCAAs daily) improved liver disease scores and other indicators of liver cirrhosis severity (Park et al., 2017). Randomized control trials have also found improvements in general health scores (Marchesini et al., 2003), fatigue (Nakaya et al., 2007), and sleep disturbances (Ichikawa et al., 2010) following BCAA supplementation. Further, BCAA supplementation improves muscle strength (Tajiri and Shimizu, 2013), increases protein metabolism and suppresses further worsening of symptoms (Togo et al., 2005). Additionally, patients with liver cirrhosis also develop insulin resistance (Kawaguchi et al., 2011). However, both intravenous (Tabaru et al., 1998) and oral (Korenaga et al., 2008) BCAA supplementation has been found to reduce blood glucose levels. This literature highlights the therapeutic potential of BCAA supplementation in limiting the severity of symptoms and outcomes in liver cirrhosis patients.

Hyperammonemia is another cause of reduced BCAAs in liver cirrhosis patients (Holecek et al., 2011). Following hyperammonemia, skeletal muscle can absorb ammonia from the blood and detoxify it via synthesis of glutamine, a process known as ammonia detoxification (Holecek et al., 2011). In slow twitch soleus muscles, hyperammonemia reduces (∼80%) BCAA (leucine, isoleucine, and valine) release and increases (∼1.3-fold) BCKA (KIV, KIC, and KMV) release. Similarly, in fast twitch EDL, hyperammonemia reduced BCAA release (60%), increased BCKA release (∼160%) and increased leucine oxidation (∼1.7-fold). In addition, AAs glutamine, glutamic acid and alanine are all reduced following hyperammonemia in the soleus and EDL. Interestingly, these alterations in BCAA catabolism had no effect on protein synthesis, protein turnover or myofibrillar proteolysis in skeletal muscle (Holecek et al., 2011). However, studies in patients with liver cirrhosis have found BCAA supplementation to improve muscle strength (Tajiri and Shimizu, 2013), increase protein metabolism and suppress further worsening of liver cirrhosis (Togo et al., 2005). These beneficial effects of BCAA are contrary to what has been described about the link between BCAA and insulin resistance/T2DM and show that the effects of BCAA on health outcomes are context-dependent.

### Chronic Renal Failure

Chronic renal failure (CRF) is associated with decreased plasma and skeletal muscle levels of the BCAAs and BCKAs (Indo et al., 1987). In addition, metabolic acidosis, a common condition found in CRF patients, is also linked to increased abundance of the BCKDH complex in muscle (Hara et al., 1987). CRF patients display elevated BCAA oxidation in skeletal muscle, which may serve to provide nitrogen for glutamine production and excretion of acids in the kidney (Mitch et al., 1994). Thus, increased abundance and activity of BCKDH in skeletal muscle is primarily responsible for the decreased levels of BCAAs and BCKAs found in CRF. Interestingly, ingestion of AAs in CRF patients increases uptake of the non-essential AAs, but not of the BCAAs in skeletal muscle. Therefore, restricted protein intake along with BCAA supplementation may be required to delay renal disease progression and uremic toxicity (Cano, 2009; Holeček, 2018). Since the BCKAs are also reduced in CRF (Indo et al., 1987), ketoanalogues of AAs and of BCAAs are also often used in the treatment of CRF. Twelve to 24 weeks of ketoacid treatment enhanced renal function, exemplified by decreased proteinuria, glomerular sclerosis, and tubulointerstitial fibrosis (Gao et al., 2010; Meisinger et al., 1987; Zhang et al., 2016; Liu et al., 2018). Previous studies have reported similar findings (Maniar et al., 1992), in addition to enhanced globular filtration rate and survival in rats (Barsotti et al., 1988). More recently, a study by Wang et al. found that 24 weeks of ketoacid treatment in rats with CRF increased body weight, mitochondrial electron transport chain activity and decrease oxidative damage in muscle (Wang D. et al., 2018). The mechanisms of beneficial effects of supplementation with ketoacids on muscle require further investigation. While the mechanisms may involve BCKA to BCAA conversion, the beneficial effects may also be independent of BCAA, for example by the actions of the ketoacids on mitochondrial integrity and function.

### Maple Syrup Urine Disease

Maple syrup urine disease (MSUD) is a hereditary recessive disorder characterized by neurological and development dysfunction, and by a distinct sweet odor in the urine of infants (Indo et al., 1987). Pathogenesis of MSUD is linked to impairments in BCAA catabolism, most often due to alterations in the BCKDH complex. MSUD is classified as types I, II, or III depending on whether E1, E2, or E3 subunit of BCKDH is mutated (Nobukuni et al., 1991). Individuals with type 1A MSUD present with a missense mutation at the C-terminal aromatic residue of the E1α subunit of the BCKDH assembly. This mutation hinders the catalytic activity of BCKDH (Menkes et al., 1954). Similarly, an 11-bp deletion mutation in the E1β subunit of the BCKDH complex that results in a change to the reading frame and subsequent E1α instability, also causes a decrease in enzymatic function *in vitro* (Nobukuni et al., 1991). These findings suggest the importance of E1β in normal functioning of the BCKDH complex in MSUD.

Evidence of abnormalities in skeletal muscle are also found in MSUD patients. Muscle fibers from MSUD patients show abnormal myofibril physiology (Ferrière et al., 1984). In addition, decreases in fiber cross sectional area (CSA) of the quadriceps (∼30%) and gastrocnemius (35%) are observed in MSUD mice. These mice show intra-muscular build up of AMP and NADH, suggesting mitochondria dysfunction as a result of BCKA accumulation, especially KIC (Sonnet et al., 2016). Accumulation of NADH in this condition is another point of link between BCAA catabolism and fatty acid metabolism, as NADH would affect the TCA cycle and impede β-oxidation of fatty acid. The data also suggest that upregulating BCKDH activity by treatment with, for example BT2, an inhibitor of BDK (Neinast et al., 2018), may attenuate accumulation of BCKAs in MSUD.

### Sepsis and Trauma Injury

Previous reports have suggested that sepsis and trauma [which often lead to muscle wasting (Owen et al., 2019)] is associated with increased BCAA oxidation in skeletal muscle (Holecek and Sispera, 2014). Mechanistically BCAAs are an important nitrogen donor for glutamine synthesis (Holecek and Sispera, 2014). Glutamine is a conditionally essential AA (Newsholme and Hardy, 1997) that is required for, amongst others, functioning of the immune system (Exner et al., 2002). Due to the reduction in glutamine pools in sepsis and trauma, the skeletal muscle compensates to increase glutamine production and does so through enhanced BCAA oxidation (Roth et al., 1982 and Holecek and Sispera, 2014). This is consistent with an increase in BCKDH activity in sepsis (Holeček, 2018). Also, increased inflammatory cytokines in sepsis decrease BCAA absorption from the gut and inhibit BCAA transport from the blood to muscles (Hasselgren et al., 1988; Gardiner and Barbul, 1993). Leucine release from the liver of endotoxin-treated animals after addition of KIC supplementation (Holecek et al., 1998) suggests that visceral tissues aminate BCKAs to produce BCAAs in sepsis in response to the reduced transport of BCAAs to the muscle from the blood.

### Type 2 Diabetes/Obesity

As discussed in Section “Diabetes/Insulin Resistance,” BCAA catabolism in skeletal muscle, liver, and adipose tissue is reduced in T2DM. BCKDHβ (∼50%) and BCAT2 (∼25%) mRNA expressions are reduced in skeletal muscle of type 2 diabetic subjects (Hernández-Alvarez et al., 2017). However, db/db mice (a model of diabetes) display no changes in BCKDHα or BCKDHβ mRNA levels, but show a decrease (∼50%) in BCKDHα protein expression and an increase in BCAT2 protein levels (∼25%) in skeletal muscle (Hernández-Alvarez et al., 2017). Similar alterations are seen in liver and adipose tissue. Liver BCKDH activity (∼2-fold), and adipose tissue BCKDHα (∼90%) and BCAT2 (∼90%) protein levels are also reduced in db/db mice (Hernández-Alvarez et al., 2017). Reduced abundance of BCKDH is consistent with suppressed (∼40%) BCKDH activity in skeletal muscle of type 2 diabetic mice. Concomitantly, BDK protein expression is ∼2-fold greater, with no change in PP2Cm (Lian et al., 2015). Furthermore, BCAA levels are elevated in the hearts of diabetic mice (twofold). These mice exhibit a defect in BCAA catabolism by way of reduced PP2Cm expression (∼40%) and diminished BCKDH activity in the heart (Lian et al., 2020). Interestingly, these changes appear specific to diabetes, as ob/ob mice show no change in the mRNA or protein expression of BCAT2 or BCKDH in skeletal muscle (Hernández-Alvarez et al., 2017). In another study, ob/ob mice exhibit significant decreases in BCAT2, BDK, PP2Cm, and BCKDH mRNA expression in adipose tissue (Zhou et al., 2019), but to a lesser extent for BDK. Downstream BCAA catabolic enzymes are also downregulated in ob/ob mice adipose tissue. This is also seen in the liver, but the effect is not as drastic, and BDK expression is increased (∼50%). On the other hand, in skeletal muscle of ob/ob mice, there is no change in the mRNA expression of these BCAA catabolic enzymes (Zhou et al., 2019). This differential tissue regulation of these enzymes results in increased BCAAs in adipose tissue, a decrease in liver BCAAs and no change in skeletal muscle. This suggests that obesity primarily targets BCAA catabolism in adipose tissue and liver but not in skeletal muscle. However, glycolysis, a process that converts glucose into pyruvate may induce BCAA catabolism in skeletal muscle. Glycolysis increases TCA cycle flux and the supply of α-KG for BCAT2. It is also possible that in insulin resistance, impairment in glucose metabolism may be linked to altered BCAA catabolism. Under such a condition, glycolysis is reduced, resulting in reduced pyruvate, and ultimately less TCA cycle flux and α-KG for transamination of BCAAs by BCAT2 (Holeček, 2020). This leads to reduced BCAT2 activity in skeletal muscle, and increased BCAA levels in plasma. Related to this, treatment with a BDK inhibitor BT2, which increases BCAA catabolism, restores the decrease in insulin sensitivity normally associated with elevated BCAA/BCKAs, suggesting that increasing BCAA catabolism may help against obesity-induced insulin resistance (Zhou et al., 2019). She et al. (2007b) support this, as in an animal model of obesity, plasma BCAA levels were ∼50% greater in Zucker rats compared to lean mice. Interestingly, no changes were found for the protein content of either BCAT2 or BCKDH E1α in the gastrocnemius. However, these mice exhibit reduced BCKD E1α protein content in both the liver (∼30%) and adipose tissue (∼60%) (She et al., 2007b). On the other hand, one study showed significant decreases in BCKDH activity (∼40%) in skeletal muscle of ob/ob mice, consistent with a significant increase in BDK protein levels (∼2-fold), but no change in PP2Cm protein levels (Lian et al., 2015). Although the studies reviewed do not all show a consistent pattern of regulation of BCAA catabolism enzymes in muscle, they all point to altered tissue and whole-body metabolism of these AAs in obesity/insulin resistant states, and that correcting such defects ameliorates insulin resistance.

### Skeletal Muscle Wasting and Fatigue

Although not a disease, altered BCAA metabolism is seen in skeletal muscle wasting and fatigue. There is an accumulation of muscle inosine monophosphate (IMP) in BCAT2 KO mice. This finding is likely due to the decreased exercise tolerance and increased muscle fatigue in these mice (She et al., 2010). However, BCAT2 KO mice fed a BCAA diet show a 39% increase in protein synthesis. The increased protein synthesis correlates with elevated mTORC1 signaling, indicated by higher 4E-BP1 and S6 phosphorylation. Interestingly, these mice present without any change in muscle weight and structure (She et al., 2007a). These results suggest that disruption of BCAA metabolism (by way of BCAT2 KO) may not only promote muscle fatigue and decreased exercise tolerance (She et al., 2007a, 2010), but may also upregulate pathways involved in both skeletal muscle protein synthesis and breakdown (Lynch et al., 2015).

## Effects of Altered/Disrupted BCAA Catabolism on Whole-Body Metabolism

### Insulin Resistance/Type 2 Diabetes Mellitus

BCAAs are nutrient signals required for muscle protein synthesis and growth (Yoshizawa, 2004). However, elevated levels of plasma BCAAs are seen in insulin resistant disorders such as obesity (Newgard et al., 2009; Lackey et al., 2013; Mccormack et al., 2013) and T2DM (Andersson-Hall et al., 2018; Vanweert et al., 2020). T2DM patients also exhibit elevated levels of the BCAAs (∼13%) in skeletal muscle (She et al., 2013) and plasma BCKAs are higher in insulin resistant individuals (Newgard et al., 2009; Mccormack et al., 2013; Giesbertz et al., 2015). Increased BCAA levels in skeletal muscle can lead to sustained mTORC1/S6K1 activation and thus impair insulin signaling/sensitivity via attenuation of PI3K/Akt signaling (Yoon, 2016; Crossland et al., 2020). This is evident as leucine (150 μM) treatment in starved L6 myotubes suppresses insulin-stimulated glucose uptake (∼37%), with an increase in S6K1 phosphorylation (∼30%). KIC (200 μM), the ketoacid of leucine, too suppresses insulin-stimulated glucose uptake (∼60%) (Moghei et al., 2016; Mann and Adegoke, 2021), with an increase in S6K1 phosphorylation (∼5-fold) (Moghei et al., 2016). This effect of KIC is attenuated in BCAT2-depleted cells (Mann and Adegoke, 2021), suggesting that KIC is converted back to leucine to suppress insulin-stimulated glucose uptake. In line with this, myotubes depleted of BCKDH have reduced insulin-stimulated glucose uptake (∼33%), but with no change in the Thr389 phosphorylation of S6K1, suggesting an alternative mechanism of insulin resistance (Mann and Adegoke, 2021). For example, increased levels of 3-hydroxyusobutyrate (3-HIB), a metabolite of valine, may contribute to insulin resistance. Knockdown of 3-HIB dehydrogenase, the enzyme that catabolizes 3-HIB, results in 3-HIB accumulation. This leads to increased endothelial fatty acid uptake by the skeletal muscle, contributing to reduced insulin sensitivity (Jang et al., 2016). Interestingly, BCAA restricted Zucker fatty rats show a reduced level of malonyl CoA, a regulator of fatty acid oxidation (Ruderman et al., 1999; McGarrah et al., 2020), potentiating increased insulin sensitivity (Ruderman et al., 1999). Restricting BCAAs in Zucker rats improves skeletal muscle insulin sensitivity, along with enhanced fatty acid oxidation and acyl-glycine export (White et al., 2016). On the other hand, body builders and athletes that consume BCAA supplements are not insulin resistant (Helms et al., 2014; Shou et al., 2019). This suggests that the effects BCAA consumption on insulin sensitivity likely depend on physiological context (energy and substrate requirements) and underlying (subtle) co-morbidities. For example, as discussed above (see section “Exercise”), exercise increases BCAA catabolism (Wagenmakers et al., 1989; Shimomura et al., 1995; Fujii et al., 1998) and improves insulin resistance, which many offset the increase in BCAA consumption by these athletes.

As discussed in Sections “Nutritional Regulation of BDK” and “PP2Cm Structure,” BDK and PP2Cm regulate ATP-citrate lyase (White et al., 2018). These findings suggests a link between increased BCAA catabolic flux and fatty acid uptake/metabolism in the development of insulin resistance (Jang et al., 2016). Consistent with this, mice heterozygous for the BCAA catabolic enzyme methylmalonyl-CoA mutase exhibit insulin resistance and reduced fatty acid oxidation (Lerin et al., 2016). This could be because reduced BCAA oxidation results in a reduced availability of BCAA-derived anaplerotic TCA intermediates, which has been shown to reduce fatty oxidation and increase the accumulation of acylcarnitines that can activate proinflammatory pathways (Adams et al., 2009), contributing to insulin resistance. In line with this, treatment with BT2 (an inhibitor of BDK) and adenoviral expression of PPM1K significantly increase insulin sensitivity via enhanced BCKDH activity and improvement in lipid metabolism (White et al., 2018).

### Heart Failure/Cardiovascular Diseases

Pathological cardiac hypertrophy is an indicator of heart failure. In cardiomyocytes, the transcription factor KLF15 inhibits hypertrophic gene expression and protein synthesis (Fisch et al., 2007). In addition, KLF15 is a central regulator of BCAA metabolism in cardiomyocytes (Fan et al., 2018). Ablation of KLF15 in mice reduces mRNA (by 50–75%) and protein (∼50%) expression of known BCAA catabolic genes, BCAT2, BCKDHα, BCKDHβ and PP2Cm in the heart. The BCAA catabolic defect in these mice led to an impairment in cardiac contractility and greater susceptibility to heart failure. On the other hand, stimulation of BCAA catabolism by BT2 increases cardiac BCKDH activity (∼7-fold) and reduces plasma BCKA levels (Sun et al., 2016). Consistent with this observation, stimulation of BCAA catabolism in cardiomyocytes that mitigates accumulation of BCAAs/BCKAs maintains cardiac function and increases the life span of cardiomyocytes (Tso et al., 2013).

### Autism and Neurological Disorders

Autism Spectrum Disorder (ASD) is a neurodevelopment abnormality characterized by an impairment in social interaction (Sparks et al., 2002). Previous reports have suggested causative links between impaired BCAA metabolism and ASD. In one study, deleting an AA transporter (solute carrier transporter 7A5) in the brain results in abnormal AA profiles and neurological abnormalities in mice (Tǎrlungeanu et al., 2016). In autistic patients, homozygous nonsense mutations in BDK results in reduced phosphorylation of the E1α subunit of BCKDH, leading to decrease in plasma BCAA levels (Novarino et al., 2012). Similarly, whole-body BDK knockout in mice increases brain levels of AAs (threonine, phenylalanine, tyrosine, histidine, and methionine) and causes abnormal neurobehavior, along with reduced BCAA levels. Interestingly, BCAA supplementation reverse the increased levels of neural AAs, the neurological defects and normalizes plasma BCAA levels (Novarino et al., 2012).

### Cancer

Different cancer types show alterations in BCAA metabolism (Sivanand and Vander Heiden, 2020). A common feature of many tumor cells is an increased requirement for glutamine (Yang et al., 2017; van Geldermalsen et al., 2018; Jiang et al., 2019; Matés et al., 2019), an AA that can be produced via amino group donation from the BCAAs (Holecek and Sispera, 2014). Some tumor cells can use BCAAs as alternative ‘fuel’ to drive tumor formation (Keenan and Chi, 2015). Overexpression of BCAT1 in mice accelerates tumor growth in hepatocellular carcinoma (Zheng et al., 2016), breast carcinoma (Siddiqui and Williams, 1989), endometrial cancer (Wang P. et al., 2018), and myeloid leukemia (Hattori et al., 2017), while suppression of BCAT1 reduces proliferation in glioblastoma (an aggressive cancer of the brain and spinal cord) (Tönjes et al., 2013).

In hepatocellular carcinoma, BCAT1/2 mRNA is elevated. However, BCKDH and other BCAA catabolic enzymes are decreased (Ericksen et al., 2019). Mechanistically, BCKAs would be unable to undergo further metabolism, leading to re-amination into BCAAs and subsequent increases in mTORC1 activity. Consistent with this, overexpression of BCAT1 in hepatocellular carcinoma leads to chemoresistance following cisplatin treatment (Zheng et al., 2016).

In breast cancer, BCAT1 and BCKDH are upregulated, enhancing BCAA metabolism and leading to increased substrates for the TCA cycle. BCAT1 knockdown resulted in blunted growth of breast cancers (Zhang and Han, 2017). In addition, expression of BCAT1 activates mTORC1 signaling to increase mitochondrial biogenesis and ATP production contributing to growth and colony formation of breast cancer cells (Zhang and Han, 2017). Similarly, tumors from ovarian cancer patients also have upregulated BCAT1. Mechanistically, inhibition of BCAT1 may not only reduce genes associated with tumorigenesis, but may also result in less energy production from the TCA cycle (Wang et al., 2015).

Elevated BCAA levels are linked to promotion of tumor development in patients with PDAC (Lieu et al., 2020). In addition to other possible roles, PDAC also utilizes BCAAs as a carbon source for fatty acid biosynthesis during cell proliferation (Lee et al., 2019). Consistent with this data, increased BCAT2 and BCAA catabolism are seen in PDAC, and knockout of BCAT2 prevented PDAC development (Li J. T. et al., 2020), highlighting the necessity for BCAT2-mediated catabolism in PDAC. However, a previous report did not demonstrate a role for BCAT1/2 in PDAC (Mayers et al., 2017) suggesting the need for more research in this area. In sum, inhibition of BCAT seems to hold promise for treatment of multiple cancers.

In addition to the impact of altered BCAA catabolism on metabolic diseases such as insulin resistance and its sequelae (T2DM, cardiovascular disease), as well as some cancers, the studies reviewed here show that impairments in BCAA catabolism have implications for neurological disorders.

## Concluding Comments and Outstanding Questions

A major issue in this discussion is whether the altered metabolism of BCAA in muscle and other tissues cause or are a consequence of the associated diseases. Except in relatively rare conditions such as MSUD, where impairments in BCAA catabolism are causative, it is challenging to prove causality between BCAA catabolism and the indicated human metabolic diseases. Studies in rodents, which permit use of specific inhibitors and genetic perturbations, have proven useful. Replicating such studies in humans will be more challenging if not impossible. As mentioned before, data on the beneficial effects of protein and BCAA supplementation in athletes clearly indicate that the effects of these nutrients on metabolic outcomes are context dependent. It may not be possible to correct for all the metabolic confounders because, for example, the effects of such confounders might be subtle and additive, even if not measurable because they are at initial stages of impairments.

The link between BCAA catabolism and lipid metabolism suggest that dietary interventions that focus on one of these macronutrients at the expense of other are likely to yield an incomplete picture. Related to this, consumption of whole egg promotes greater myofibrillar protein synthesis compared to egg white (which is a complete, high quality protein) in men, post resistance exercise (Van Vliet et al., 2017). In that study, energy intake was not balanced between study groups but the links between BCAA catabolism and lipid oxidation reviewed here would suggest that the difference might not be due to caloric content of the two foods alone. Related to this is how the nature of the fatty acids (mono or polyunsaturated fatty acids, *n* − 3 vs. *n* − 6) would modulate the effect of protein/AA supplements in athletes.

The anabolic effects of leucine and other BCAAs are least in part linked to their effects on mTORC1. Interestingly, insulin stimulates BCAA catabolism (Neinast et al., 2018). Moreover, insulin resistance is linked to both altered BCAA catabolism (Lerin et al., 2016; Wang et al., 2019) and increased mTORC1 signaling (Moghei et al., 2016; Yoon, 2016). An intriguing question then is whether mTORC1 regulates BCAA catabolism. White adipocytes from adipose tissue-specific raptor knockout (i.e., attenuated mTORC1 signaling) mice are impaired in their ability to respond to PPARγ-induced catabolism of BCAA (Andrade et al., 2021). In addition, leucine-induced increase in heart BCKDH complex activity is impaired in the presence of rapamycin, an inhibitor of mTORC1 (Zhen et al., 2016). This effect of mTORC1 on BCAA catabolism appears counter-intuitive, given the anabolic nature of the complex. We are unaware of similar studies in skeletal muscle but if mTORC1 activates muscle BCAA catabolism, this would be inconsistent with the beneficial effects of rapamycin in ameliorating insulin resistance (Leontieva et al., 2014; Blagosklonny, 2019).

While the three BCAA have anabolic properties, an emerging concept is the observation that each of these AAs has unique metabolic imprints. For example, a recent study shows that many of the metabolic abnormalities attributed to BCAA are mediated by isoleucine and to a lesser extent valine, with leucine having little to no effect (Yu et al., 2021). Although another recent study in C2C12 did not find an effect of valine on insulin resistance (Rivera et al., 2020a), the data from Yu et al. (2021) are consistent with a previous report of greater effect of valine (compared to leucine) in inducing insulin resistance of glucose transport in L6 myotubes (Tavajohi-Fini, 2012) and of the effect of the valine metabolite 3-hydroxyusobutyrte (3-HIB) in inducing insulin resistance in skeletal muscle (Jang et al., 2016). Because normally, humans do not consume these AAs singly in isolation, it will be interesting to see the relevance of this in management of insulin resistance. In addition, because the three BCAA are essential AAs that are required in relatively large amounts, if the data on the impact of each of them on metabolic abnormalities are confirmed in other human studies, this would most certainly have implications for their requirements. For example, how would consuming a diet perpetually low in isoleucine and valine affect tissue (especially skeletal muscle) protein metabolism and muscle mass?

Aging is associated with increased rate of muscle loss and strength (sarcopenia) which can be improved with essential AA ingestion and resistant exercise (Dickinson et al., 2013). Also, for many of the human diseases, including diabetes, cardiovascular disease, insulin resistance and cancer, age is the most important risk factor (Hall, 2015; Mullard, 2018). How BCAA catabolism, specifically the activities of BCAT2 and BCKDH, change with age is an area that needs to be explored. Finally, as mentioned before, not much is known about the post translational regulation of the BCAA catabolic enzymes in skeletal muscle.

There is growing literature suggesting that diseases such as T2DM, insulin resistance, renal failure and MSUD develop in response to altered whole-body and skeletal muscle BCAA metabolism. Future studies addressing gaps in our understanding of the links between altered metabolism of the BCAAs and muscle and whole-body physiology are warranted. From such studies, we may identify metabolic targets that can be explored and used in the prevention/treatment of many human diseases, especially those that lead to defects in muscle growth and functions.

## Author Contributions

GaM, SM, and GlM drafted the initial version of the manuscript. GaM, SM, GlM, and OA reviewed and edited the manuscript. All authors approved the final version of the manuscript.

## Conflict of Interest

The authors declare that the research was conducted in the absence of any commercial or financial relationships that could be construed as a potential conflict of interest.

## Abbreviations

3-HIB: 3-hydroxyusobutyrate
4E-BP1: eukaryotic mRNA translation initiation factor 4E-binding protein 1
α-KG: α-ketoglutarate
AA: amino acids
AKT: protein kinase B
AMBRA1: autophagy and beclin 1 regulator 1
ASD: autism spectrum disorder
APN: adiponectin
ATG13: autophagy-related protein 13
BCAA: branched-chain amino acids
BCAT: branched-chain aminotransferase
BCAT1: cytosolic BCAT
BCAT2: mitochondrial BCAT
BCKDH: branched-chain α-keto acid dehydrogenase complex
BT2: 3-chloro-benzo[b]thiophene-2-carboxylic acid
CHREBP- β: carbohydrate response element binding protein
CRF: chronic renal failure
DLD: dihydrolipoamide dehydrogenase
E1: heterodimeric branched-chain α-keto acid decarboxylase
E2: dihyrolipoyl transacyclase homodimeric
E3: dihydrolipoyl dehydrogenase
GLN: glutamine
GR: glucocorticoid receptor
HEK293: human embryonic kidney 293 cells
HFD: high-fat diet
HMB: beta-hydroxy-beta-methylbutyrate
IGF1: insulin like growth factor 1
IMP: inosine monophosphate
IRS1: insulin receptor substrate 1
KIC: 2-ketoisocaproate/4-methyl-2-oxopentanoic acid
KLF15: kruppel like factor 15
KMV: α-keto - β-methylvaleric acid/3-methyl-2-oxopentanoate
KO: knockout
KIV: 2-keto-isovalerate/3-methyl-2-oxobutanoic acid
MIR: myocardial ischemia reperfusion
miRNA: microRNAs
mKO: muscle knockout
MSUD: maple syrup urine disease
mTORC1: mammalian target of rapamycin complex 1
mPK: mitochondrial protein kinase
PDAC: pancreatic ductal adenocarcinoma
PGC-1 α: peroxisome proliferator-activated receptor gamma coactivator-*1alpha*
PHK: protein histidine kinase
PI3K: phosphatidylinositol-3 kinase
PLP: pyridoxal 5 ′ -phosphate
PMP: pyridoxamine 5 ′ -phosphate
PP2CM: mitochondrial protein phosphatase 2 cm
PPAR α: peroxisome proliferator activated receptor alpha
PPM1K: protein phosphatase mg^2+^/mn^2+^ dependent 1K
ROS: reactive oxygen species
S6K1: ribosomal protein S6 kinase
T1DM: type 1 diabetes mellitus
T2DM: type 2 diabetes mellitus
TFEB: transcription factor EB
T3: thyroid hormone (3,5,3 ′ -triiodothyronine)
TNF α: tumor necrosis factor alpha
TRIM21: tripartite motif containing-21
UBE3B: ubiquitin E3 ligase
ULK-1: unc-51 like autophagy activating kinase
UPP: ubiquitin proteasome pathway.
